# Calcium sensing receptor involving in therapy of embryonic stem cell transplantation alleviates acute myocardial infarction by inhibiting apoptosis and oxidative stress in rats

**DOI:** 10.22038/ijbms.2020.47436.10916

**Published:** 2020-10

**Authors:** Hui Yuan, Guohong Yang, Shu Li, Li Li, Tao Wei, Gaochen Song, Hairong Luan, Jin Meng, Qi Wang, Yaquan Yu, Jian Sun

**Affiliations:** 1 Mudanjiang Medical University, Mudanjiang, 157011, China

**Keywords:** Acute myocardial infarction, Apoptosis, Calcium sensing receptor, Cell transplantation, Embryonic stem cell

## Abstract

**Objective(s):**

The aims of the present study were to investigate the expression of calcium sensing receptor (CaSR) at different times in acute myocardial infarction (AMI) rat myocardial tissue after mouse embryonic stem cells (mESCs) transplantation treatment and to assess its effects on apoptosis and oxidative stress of cardiomyocytes.

**Materials and Methods:**

The AMI rats were treated with mESCs, Calindol (a CaSR agonist) and Calhex231 (a CaSR inhibitor). Serum measurements, Echocardiographic analysis and TUNEL assay were performed. Myocardial ultrastructure changes were viewed by electron microscopy. Additionally, western blotting was used to detect the protein expressions.

**Results:**

Compared to the sham group, it was found that the expression levels of CaSR, caspase-3, cytoplasmic cytochrome C (cyt-C) and Bcl2-associated x (Bax), and the levels of Malondialdehyde (MDA) were significantly increased in both AMI and AMI + mESCs + Calindol groups with the development of myocardial infarction. Furthermore, the ultra-microstructure of cardiomyocyte was highly damaged, the expression levels of mitochondrial cyt-C and B-cell lymphoma 2 (Bcl-2) were significantly decreased, and there was decreased activity of superoxide dismutase (SOD). However, the combination of Calhex231 and mESCs transplantation could inhibit these changes.

**Conclusion:**

Our results suggested that CaSR expression in myocardial tissue of AMI rats was increased over time, and that Calhex231 could enhance the efficacy of ESCs transplantation for the treatment of AMI, which would be a new therapeutic strategy for the treatment of AMI.

## Introduction

Acute myocardial infarction (AMI) is the myocardial necrosis caused by acute and persistent ischemia and hypoxia of coronary artery. AMI can cause irreversible damage and loss of cardiomyocytes, which can lead to arrhythmia and heart failure (1). On the other hand, there are few effective drugs to repair the infarct areas currently. In recent years, cell therapy has provided a promising strategy for the treatment of AMI. Various types of stem cells with differentiation potential are transplanted into myocardial infarction areas, and then differentiated into cardiomyocytes to replace the missing myocardial cells and improve the heart functions (2). Nevertheless, the exact mechanism underlying AMI is still not completely understood, which weakens the effects of cell therapy methods (3). 

The calcium sensing receptor (CaSR) is a G-protein coupled receptor with seven transmembrane domains that plays a role in regulating intracellular calcium concentration, and participates in cell differentiation, apoptosis, gene expression, membrane potential and ion channel switching (4, 5). The expression of myocardial CaSR in healthy rats and rats with myocardial ischemia/reperfusion injury has been observed in previous studies (6-9). However, the variations in CaSR expression in different phases of AMI rats and its effects on cardiac function have not been elucidated. 

The present study established an AMI model by coronary artery ligation to observe the changes of CaSR expression at different time periods. Moreover, mouse embryonic stem cells (mESCs) transplantation therapy, CaSR agonist and CaSR inhibitor treatments were used to assess the levels of serum myocardial enzymes, cardiac function, myocardial histomorphology, myocardial oxidative stress and the degree of apoptosis. The aims of the study were to investigate the functional role and identify possible mechanisms of CaSR involved in the treatment of mESCs transplantation in AMI rats. 

## Materials and Methods

***Materials***

The ES cell line (CRL-11632) was purchased from the American Type Culture Collection. cyt-C (sc-130311), Bcl-2 (sc-7382), Bax (sc-493), caspase-3 (sc-7148), Calindol (sc-211006) and Calhex231 (sc-207394) were purchased from Santa Cruz Biotechnology, Inc. CaSR (ab137408) was purchased from Abcam. TUNEL kit (s7110) was purchased from EMD Millipore. WET-1 method for determination of SOD kit (s0103) and malondialdehyde (MDA) assay kit (s0131) were from Beyotime Institute of Biotechnology.

***Acute myocardial infarction model***

All experiments conformed to the Guide for the Care and Use of Laboratory Animals published by the China National Institutes of Health. The protocol was approved by the Mudanjiang Medical University Medical Science Ethics Committee. Healthy Wistar rats (weight, 200-220 g; age, 8 weeks) were maintained on a 12-hr light/dark cycle and were housed in animal room of the Medical Center of Mudanjiang Medical University with pathogen-free and moderate humidity at 24-25˚C. The sterile food and water were provided and the beddings were changed in every 2 days. The rats were anesthetized by intraperitoneal injection of 50 mg/kg pentobarbital sodium(10). After complete anesthesia, the rats were intubated in a supine position and ventilated on room air via a small animal ventilator (DW-3000C, ZS-DICHUANG Co., China). The rat model of AMI was prepared by ligation of the left anterior descending branch of the coronary artery and transplantation of ESCs as previously reported (11). Animals were randomly selected for testing the behavior, health and cardiac function at weeks 1, 2 and 4 of the experiment. At the above time points, the rats were sacrificed intraperitoneally with 200 mg/kg pentobarbital sodium. After thoracotomy, the heart was quickly excised and washed with phosphate buffer to perform Western blotting and TUNEL assay. 

***Experimental groups***

In total, 80 rats were randomly divided into the following five groups (one extra rat was prepared for accidental death in each group): i) Sham group (n=16), only threading under the left anterior descending branch of the coronary artery but not ligated; ii) AMI group (n=16), the left anterior descending branch of the coronary artery was ligated; iii) AMI + mESCs group (n=16), the 129 mouse ES-D3 cells (1.6 x 10^6^ cells/0.1 ml/each point) were injected into four areas within the apical region 30 min after AMI; iv) AMI + mESCs + Calindol group (n=16), before transplanting mESCs, Calindol (CaSR agonist; 10 μmol/kg) was injected via the tail vein, and then injected every day (12); and v) AMI + mESCs + Calhex231 group (n=16), before transplanting mESCs, Calhex231 (CaSR inhibitor; 10 μmol/kg) was injected via the tail vein, and then injected every day (12).

***Serum measurements and echocardiographic analysis***

Blood samples taken from the aorta were centrifuged and serum stored at -80 ^°^C. Serum levels of creatine kinase (CK), lactate dehydrogenase (LDH), and cardiac troponin T (cTnT) were then analyzed using a standard biochemistry panel (Beyotime, Nantong, China), following the supplier’s protocols. Rats were anesthetized in an induction chamber with 3% isoflurane and maintained with 1.5% isoflurane during echocardiography examinations, as previously described (13). A Vivid 7 Dimension echocardiography machine was used to assess ST segment (GE Healthcare, Waukesha, WI, USA).

***Protein expression in myocardial tissue and mitochondria analyzed by western blotting***

Myocardial tissue and mitochondrial protein were extracted. The protein content was quantified by bicinchoninic acid method using assay kit. Then, 40 μg protein extracts of myocardial tissue and mitochondria were subjected to SDS-PAGE (10 or 14%) and blotted on PVDF membranes at 80 V for 1.5 hr. The membrane was then incubated with anti-cyt-C, anti-Bcl-2, anti-Bax, anti-caspase-3 and anti-CaSR at 37˚C for 1 hr. Subsequently, the membrane was incubated with IgG-horse radish peroxidase (HRP)-conjugated (anti-rabbit) secondary antibody at room temperature for 1 hr. Protein bands were visualized using an enhanced chemiluminescence plus western blot detection system (Immobilon Western HRP; EMD Millipore). The relative intensities of protein bands were quantified using a Bio-Rad Chemi EQ densitometer (Bio-Rad Laboratories, Inc.) and the band density was semi-quantified using AlphaView v3.2.2 software (ProteinSimple). 

***Myocardial ultrastructure changes viewed by electron microscopy (EM)***

Ultrastructural changes of rat myocardium tissue were observed by EM. Myocardial tissue of sham and experimental groups fixed in 2.5% glutaraldehyde for 2-4 hr and washed three times with phosphate-buffered saline (PBS). Tissues were then fixed in 1% osmic acid, dehydrated in ethanol, embedded and cut into ultrathin section. Sections were viewed under a transmission electron microscope (Hitachi H-7650; Hitachi Ltd.).

***TUNEL assay***

The fixed heart tissues were dehydrated, embedded in paraffin and cut into 5 μm slices. Sections were then dewaxed in water and 50 μl of 0.1% Triton X-100 (0.1% sodium citrate salt) was added. Then, sections were rinsed with PBS, and 50 μl TUNEL reaction solution was added and incubated for 60 min at 37˚C. Tissues were counterstained with hematoxylin, and observed and imaged (magnification, x400) under a microscope. 

***Statistical analysis***

All experiments were performed ≥3 times independently. Data are presented as the mean±SEM. Statistical analysis was performed by a two-tailed Student’s t-test or one-way ANOVA followed by the Bonferroni multiple comparisons test using SPSS 18.0 software (IBM Corp.). *P<0.05* was considered to indicate a statistically significant difference.

## Results

***Myocardial tissue lesion and change of serum enzyme activity***

**Figure 1:**
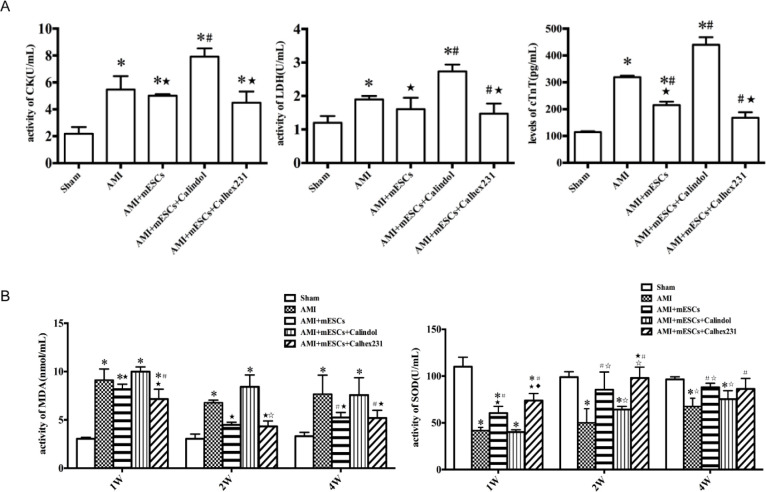
Successful modeling of AMI and oxidative stress indexes in serum. (A) CK, LDH and cTnT levels in serum (B) MDA and SOD levels in serum. *P<0.05 vs. Sham (n=5). #*P*<0.05 vs. AMI group. *P*<0.05 vs. Calindol-treated group. CK: creatine kinase, LDH: lactate dehydrogenase, cTnT: cardiac troponin T, MDA: malondialdehyde, SOD: superoxide dismutase, AMI: acute myocardial infarction

Compared to the Sham group, in the AMI group the myocardial tissue of the anterior wall of the left ventricle was pale and the contractility was decreased after the operation. It was found that the ST segment was elevated in the electrocardiogram. Furthermore, the CK and LDH activity, as well as the levels of cTnT, were significantly increased in the AMI group. There were no significant changes in CK and LDH levels after treatment with mESCs. However, Calindol could significantly enhance the CK, LDH and cTnT levels, and Calhex231 could inhibit the Calindol effects (Figure 1A).

***Changes in MDA and SOD activity in serum***

 At weeks 1, 2 and 4, compared to the Sham group, the MDA level was significantly increased in AMI group; Compared to the AMI + Calindol group, treatment with mESCs transplantation alone significantly reduced the level of MDA in serum, which was further reduced in the Calhex231 + mESCs group. However, the opposite results were observed for SOD activity (Figure 1B).

***Ultrastructural changes in the myocardial tissue of rats***

**Figure 2:**
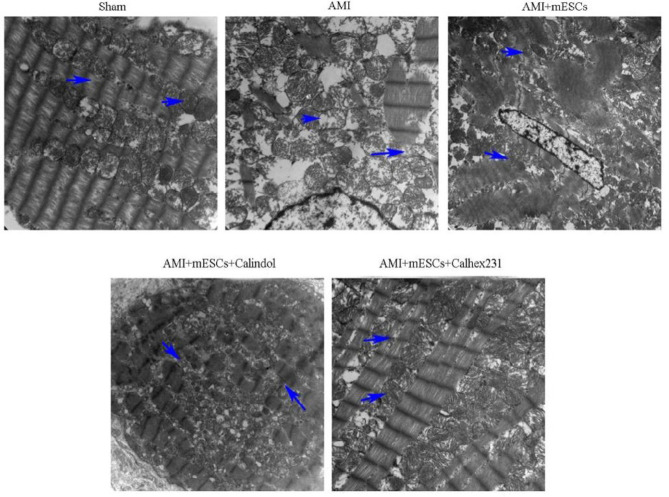
Ultrastructural changes of myocardial tissue detected by electron microscope. Magnification, x15000

Changes in the myocardial ultrastructure in rats were observed by electron microscopy. It was also identified that the nucleus and mitochondria were severely damaged. Moreover, swelling of the mitochondria, rupture of the myocardial sarcomere, disorder of arrangement and dissolution of myofibrils were observed in the AMI and Calindol-treated groups. In the AMI + mESCs and the AMI + mESCs + Calhex231 groups, the swelling of myocardial mitochondria was alleviated, the sarcomere was intact and the striation and intercalated disc were clear, especially in the AMI + mESCs + Calhex231 group. Therefore, the present results indicated that damages of myocardial cells occurred in rats after AMI, and that treatment with mESCs + Calhex231 could significantly improve these myocardial damages (Figure 2).

***The expression of calcium sensing receptor in myocardial tissue at weeks 1, 2 and 4***

**Figure 3:**
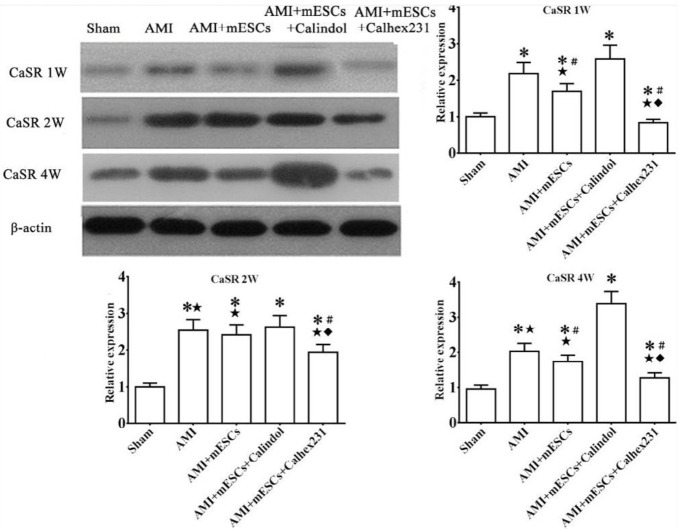
Changes in protein expression levels were detected by western blotting after AMI from week 1 to week 4. *P<0.05 vs. sham group. #*P*<0.05 vs. AMI group. *P*<0.05 vs. Calindol Hydrochloride-treated group. *P*<0.05 vs. mESC transplantation group (n=3). AMI: acute myocardial infarction, mESCs: mouse embryonic stem cells

Compared to the sham groups, the CaSR expression was increased in rat myocardial tissue with time prolongation after AMI. The CaSR expressions were increased gradually in the Calindol-treated groups at weeks 1, 2 and 4. Compared to AMI and mESCs + Calindol groups, the CaSR expression significantly decreased in mESCs + Calhex231 groups at each time points (Figure 3).

***Measurement of cardiac function***

**Figure 4:**
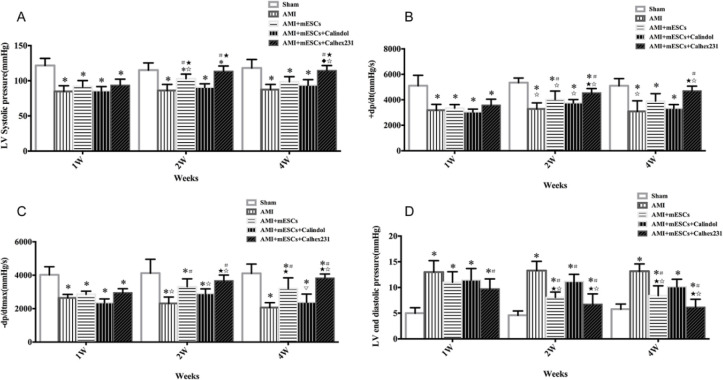
Changes of cardiac function in different group, and the effects of AMI treated with mESCs transplantation + Calhex231 at 1^st^, 2^nd^ and 4^th ^weeks

The changes of cardiac function in different groups and effect of CaSR expression on cardiac function of AMI after mESCs transplantation at weeks 1, 2 and 4 were investigated. The therapeutic effects of AMI were not obvious at 1^st^ week. At 2^nd^ and 4^th^ weeks, compared to the AMI group, left ventricular systolic pressure (LVSP; Figure 4A), maximum increase rate of left ventricular pressure (+dp/dtmax) (Figure 4B) and maximal fall rate of left ventricular pressure (-dp/dtmax)(Figure 4C) were increased in the AMI + mESCs group, and further increased in the AMI + mESCs + Calhex231 transplantation group, while left ventricular end-diastolic pressure (LVEDP) was significantly decreased (Figure 4D), suggesting that over time cardiac function was significantly improved. However, the opposite results were observed in the AMI + mESCs + Calindol groups.

***Changes in apoptosis in rat myocardial tissue***

**Figure 5:**
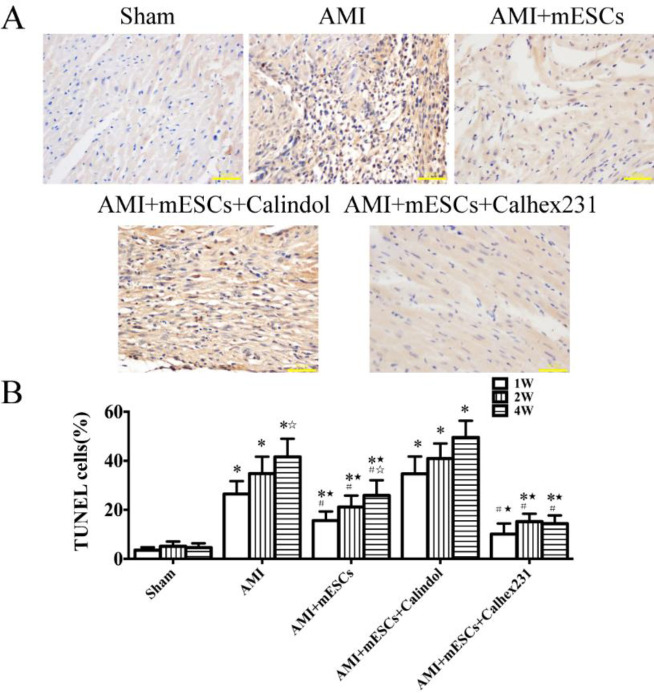
Detection of apoptosis in rat myocardial tissue by TUNEL staining. (A) Nuclei of TUNEL positive cells were brown. (B) Statistical analysis of apoptosis indexes at weeks 1, 2 and 4 in each group; Magnification, x400. *P<0.05 vs. sham group. #*P*<0.05 vs. AMI group. *P*<0.05 vs. Calindol-treated group. *P*<0.05 vs. 1^st^ week (n=5). AMI: acute myocardial infarction

At week 1, the TUNEL staining results identified that apoptosis was significantly increased in both AMI (26.48±5.22%) and Calindol-treated groups (34.73±7.06%). Furthermore, compared to the AMI and AMI + Calindol groups, the apoptotic rate of cardiomyocytes was significantly decreased in AMI + mESCs (15.65±3.69%) and Calhex231 + mESCs (10.16±4.29%) groups. From the 2^nd^ to 4^th^ weeks, the apoptotic rates in the AMI and AMI + Calindol groups were continuously increased. Moreover, compared to the AMI group, apoptosis in the AMI + mESCs group was decreased, and was further decreased in the AMI + mESCs + Calhex231 group. However, it was demonstrated that there was no significant difference at each time point. Thus, the present results suggested that AMI myocardial injury may be attenuated by combination therapy of mESCs transplantation and Calhex231 (Figure 5A and B).

***Expression levels of cytoplasmic cyt-C, Bcl-2, Bax, caspase-3 and mitochondria cyt- C in myocardial tissue at different time points***

**Figure 6:**
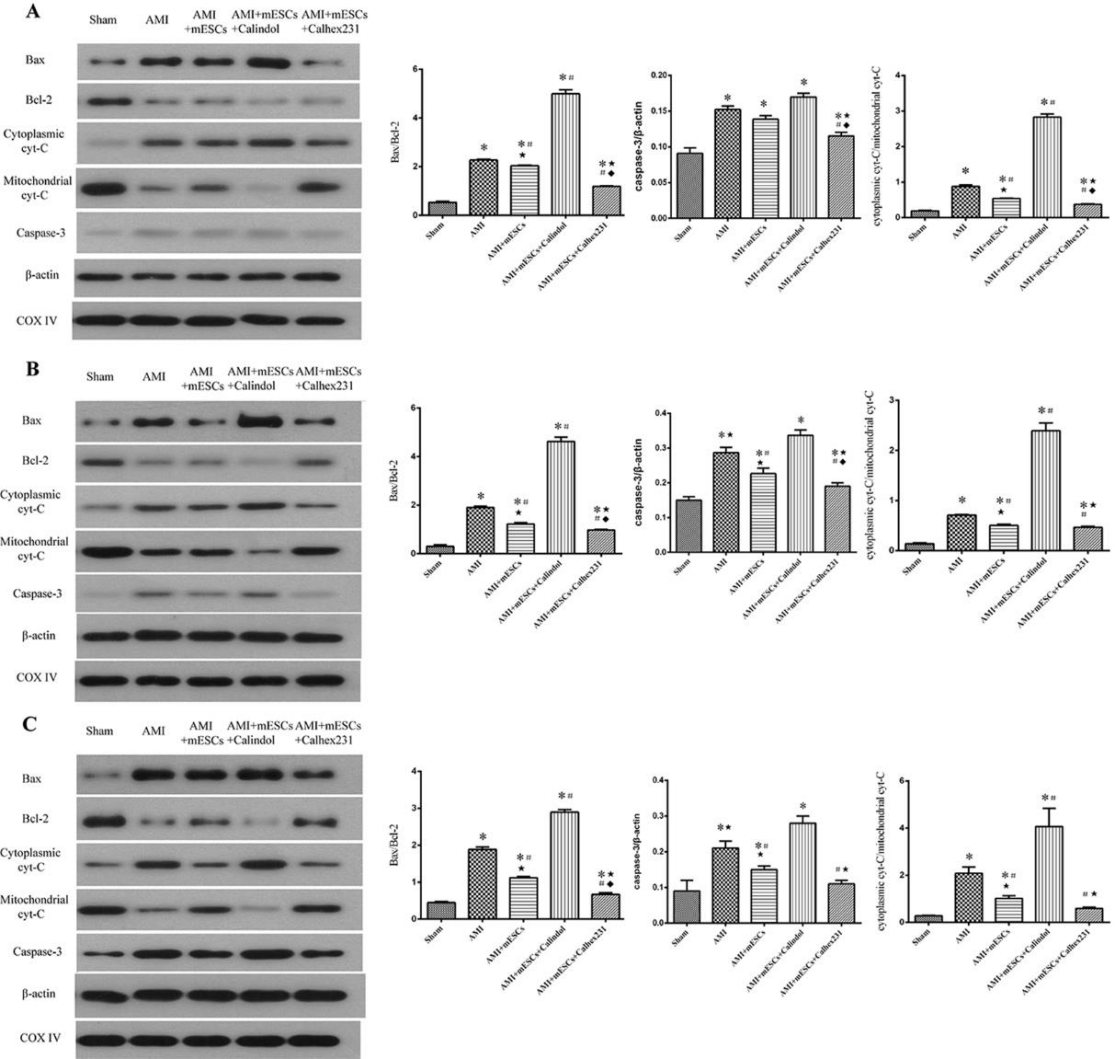
Expression of apoptotic-related proteins and cytoplasmic/mitochondrial cyt-C detected by western blotting. (A-C) Protein expression levels of cytoplasmic cyt-C, Bax and caspase-3 in the myocardial tissue of AMI rats were detected by western blotting at weeks 1, 2 and 4 in each group, respectively. *P<0.05 vs. sham group. #*P*<0.05 vs. AMI group. *P*<0.05 vs. Calindol-treated group. *P*<0.05 vs. mESCs transplantation group (n=3). AMI: acute myocardial infarction, mESCs: mouse embryonic stem cells

At weeks 1, 2 and 4, compared to the Sham group, it was found that the expression levels of the apoptosis-related proteins Bcl-2 and mitochondria cyt-C were decreased, while the expression levels of Bax, caspase-3 and cytoplasmic cyt-C were increased in the AMI group. Moreover, the expression levels of these proteins showed opposite results in the AMI + mESCs and AMI + mESCs + Calhex231 groups compared to the AMI and AMI + Calindol-treated groups. Furthermore, the changes were more significant in Calhex231-treated groups (Figure 6A-C).

## Discussion

CaSR is an important factor for regulation of calcium metabolism, which plays a role in the regulation of cell proliferation and differentiation (14). Our previous study revealed that increased expression of myocardial CaSR in atherosclerotic rats could enhance the risk of myocardial infarction (15). However, the role of CaSR at different stages after myocardial infarction or ESC transplantation treatment remains unknown. Moreover, it is not fully understood whether changes in Ca^2+^ concentration and CaSR expression can affect cardiomyocyte apoptosis and ESCs differentiation into cardiomyocytes. Therefore, an AMI rat model was established to investigate the CaSR functions. In the present study, the levels of serum LDH and CK in AMI rats were increased, which are the important markers for the degree of myocardial injury (16). cTnT level is highly sensitive and specific for diagnosis of myocardial infarction, which significantly increase in myocardial injury (17, 18). All the above-mentioned detections showed that the rats suffered from AMI. 

The electron microscopy results identified that the nucleus and mitochondria of myocardial cells were highly damaged in AMI group. Furthermore, the myocardial sarcomere fibers were disordered, the myofilaments were dissolved and broken, the mitochondria were swollen and the chromatin was concentrated; these damages were more obvious in Calindol-treated AMI rats. However, the damages of myocardial structure in AMI rats were improved after mESCs treatment, especially in mESCs + CaSR inhibitor (Calhex231) group at week 4. Collectively, the present results suggested that mESCs transplantation and Calhex231 significantly reduced the cellular damages caused by AMI, which may be related to the CaSR expression.

In order to observe the changes of CaSR after myocardial infarction at different time points, the expression of CaSR was detected by western blotting at weeks 1, 2 and 4 after establishment of AMI models. It was found that CaSR expression was gradually increased over time after myocardial infarction, and mESCs transplantation treatment could reduce the CaSR expression except in 2^nd^ week. Moreover, tail vein injection of Calhex231 or Calindol could enhance or weaken the effects of mESCs transplantation therapy; it was indicated that the increased CaSR expression was involved in the occurrence of myocardial infarction. 

Myocardial infarction is often accompanied by impaired cardiac function (19). In the present study, the changes of cardiac function were evaluated by measuring the hemodynamic indexes. LVSP, +dp/dtmax and -dp/dtmax were significantly decreased in the AMI group, while LVEDP was significantly increased. However, when the AMI rats were treated with mESCs transplantation, the aforementioned indexes were all improved. To our surprise, mESCs + Calhex231 treatment has more obvious effects, especially at weeks 2 and 4, and were closer to the normal values. Thus, it is suggested that mESCs + Calhex231 treatment may be an effective therapy for AMI.

To verify the involvement of CaSR in mESCs treatment, apoptosis and oxidative stress indexes of AMI rats were detected. The TUNEL assay results and the expression levels of apoptotic-related proteins demonstrated that the apoptosis indexes of cardiomyocytes in AMI rat were increased significantly over time, and these changes could be improved by mESCs transplantation or Calhex231; however, Calindol had the opposite effects. The production of oxygen free radicals and the degree of tissue damage can be indirectly reflected by determination of the lipid peroxidation and MDA (20). SOD is a scavenging enzyme of superoxide anion radical O^2-^, which plays an important role in the oxidation and antioxidation balance of the body (21). In the experiment, the changes of serum MDA level was in accordance with the changes of apoptosis, and the SOD activity showed the opposite trend, which indicated that underlying therapeutic mechanisms of AMI may be related to antiapoptotic effect and oxidative stress (15).

Apoptosis is an important mechanism of cardiomyocytes death during myocardial ischemia (8, 22, 23). There are two classic pathways of apoptosis, the extracellular death receptor pathway, which involves a variety of apoptotic regulatory genes such as pro-apoptotic gene Bax and anti-apoptotic gene Bcl-2, and the endogenous apoptosis pathway, which is regulated by mitochondria function (24). After myocardial infarction, mitochondrial DNA is damaged by oxidative stress reaction and leads to the enhancement of mitochondrial membrane permeability and cyt-C release into the cytoplasm, which then combines with the apoptotic protease activator (25). The two pathways act together on caspase-3, which activates downstream genes and leads to apoptosis (26). 

In the present study, it was found that the expression levels of Bcl-2 and mitochondrial cyt-C were decreased at different time points in AMI group, while the expression levels of cyt-C, Bax and caspase-3 were significantly increased, especially in mESCs + Calindol group; however, these effects were obviously inhibited by Calhex231. Therefore, it was speculated that the expression levels of apoptotic-related proteins may be regulated by CaSR activity. Collectively, with increased duration of rat myocardial infarction, the CaSR expression was gradually increased, and apoptosis and oxidative stress were increased, and these effects could be significantly enhanced by the CaSR agonist. However, these changes could be inhibited by the CaSR inhibitor and mESCs transplantation. Interestingly, the former can enhance the therapeutic effect of mESCs. Recent research data show that miRNAs or traditional Chinese medicine can enhance the therapeutic effects of mESCs transplantation, while the relevant mechanisms remain elusive (27). It is hoped that our results could provide a new research direction.

(A) LVSP. (B) + dp/dtmax. (C) dp/dtmax. (D) LVEDP. *P<0.05 vs. sham group. #*P*<0.05 vs. AMI group. *P*<0.05 vs. Calindol-treated group. *P*<0.05 vs. mESCs transplantation group. * P*<0.05 vs. 1st week. *P*<0.05 vs. 2nd week (n=5). AMI: acute myocardial infarction, mESCs: mouse embryonic stem cells, LVEDP: left ventricular end-diastolic pressure, LVSP: left ventricular systolic pressure

**Figure 7:**
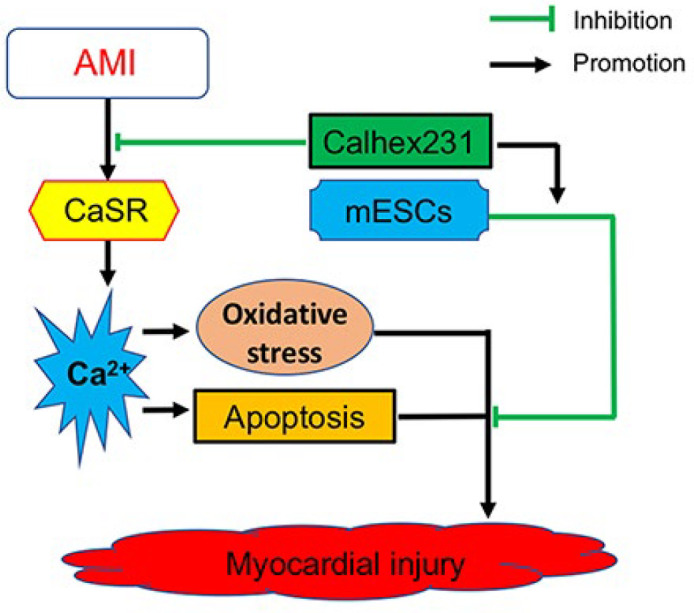
Schematic diagram illustrating the therapeutic acute myocardial infarction mechanism of Calhex231 combining with embryonic stem cell transplantation

## Conclusion

Taken together, based on both the present and previous results, it was hypothesized that the therapeutic effect of mESCs transplantation could be further enhanced by a CaSR inhibitor. The underlying mechanism of these effects may be related to reducing Ca^2+ ^concentration and reducing the occurrence of cardiomyocyte apoptosis and oxidative stress after AMI, which may be via improving the microenvironment of mESCs and promoting the differentiation of ESCs into cardiomyocytes (28), suggesting that Calhex231 combining with mESCs transplantation would be a new therapeutic strategy for the treatment of AMI (Figure 7).

## Funding Source

This work was supported by the National Natural Science Foundation of China (No. 81300163) and Basic Scientific Research Business Research Project of Heilongjiang (No. 2017-KYYWFMY-0663), and project of Mudanjiang Science and Technology Bureau (No. Z2018s049).

## Conflicts of Interest

The authors declare that there are no conflicts of interest regarding the publication of this paper.
